# Reaching the hard to reach: longitudinal investigation of adolescents’ attendance at an after-school sexual and reproductive health programme in Western Cape, South Africa

**DOI:** 10.1186/s12889-015-1963-3

**Published:** 2015-07-04

**Authors:** Catherine Mathews, Sander Matthijs Eggers, Petrus J. de Vries, Amanda J. Mason-Jones, Loraine Townsend, Leif Edvard Aarø, Hein De Vries

**Affiliations:** Health Systems Research Unit, South African Medical Research Council, PO Box 19070, Tygerberg, Cape Town 7505 South Africa; Adolescent Health Research Unit, Division of Child & Adolescent Psychiatry, University of Cape Town, 46 Sawkins Road, Rondebosch, Cape Town 7700 South Africa; Department of Health Promotion, Caphri Research Institute, Maastricht University, POB 616 6200 MD, Maastricht, The Netherlands; Division of Child & Adolescent Psychiatry, University of Cape Town, 46 Sawkins Road, Rondebosch, Cape Town 7700 South Africa; Department of Health Sciences, University of York, Seebohm Rowntree Building, Heslington, York YO10 5DD UK; Department of Health Promotion and Development, University of Bergen, Bergen, Norway; Division of Mental Health, Norwegian Institute of Public Health Division of Mental Health, P.O Box 4404, Nydalen, Oslo NOR-0403 Norway

## Abstract

**Background:**

Adolescents need access to effective sexual and reproductive health (SRH) interventions, but face barriers accessing them through traditional health systems. School-based approaches might provide accessible, complementary strategies. We investigated whether a 21-session after-school SRH education programme and school health service attracted adolescents most at risk for adverse SRH outcomes and explored motivators for and barriers to attendance.

**Methods:**

Grade 8 adolescents (average age 13 years) from 20 schools in the intervention arm of an HIV prevention cluster randomised controlled trial in the Western Cape Province of South Africa, were invited to participate in an after-school SRH program and to attend school health services. Using a longitudinal design, we surveyed participants at baseline, measured their attendance at weekly after-school sessions for 6 months and surveyed them post-intervention. We examined factors associated with attendance using bivariate and multiple logistic and Poisson regression analyses, and through thematic analysis of qualitative data.

**Results:**

The intervention was fully implemented in 18 schools with 1576 trial participants. The mean attendance of the 21-session SRH programme was 8.8 sessions (S.D. 7.5) among girls and 6.9 (S.D. 7.2) among boys. School health services were visited by 17.3 % (14.9 % of boys and 18.7 % of girls). Adolescents who had their sexual debut before baseline had a lower rate of session attendance compared with those who had not (6.3 vs 8.5, *p* < .001). Those who had been victims of sexual violence or intimate partner violence (IPV), and who had perpetrated IPV also had lower rates of attendance. Participants were motivated by a wish to receive new knowledge, life coaching and positive attitudes towards the intervention. The unavailability of safe transport and domestic responsibilities were the most common barriers to attendance. Only two participants cited negative attitudes about the intervention as the reason they did not attend.

**Conclusions:**

Reducing structural barriers to attendance, after-school interventions are likely to reach adolescents with proven-effective SRH interventions. However, special attention is required to reach vulnerable adolescents, through offering different delivery modalities, improving the school climate, and providing support for adolescents with mental health problems and neurodevelopmental academic problems.

**Trial registration:**

Current Controlled Trials ISRCTN56270821; Registered 13 February 2013.

## Background

Adolescents need to have access to proven-effective sexual and reproductive health (SRH) interventions such as comprehensive sexual health education and counselling, access to condoms, contraceptives and HIV tests, especially in sub-Saharan Africa where rates of HIV and intimate partner violence (IPV) are high [1, 2]. Yet, globally young people face a range of barriers [3] at traditional health system facilities including cost [3], transportation, clinic hours [4], privacy and confidentiality, lack of available services [5] and negative health worker attitudes [6]. Out-of-health facility approaches, which involve service provision at places where adolescents live and congregate such as youth centres and school-based approaches, are potentially important complementary strategies for increasing access to SRH services and education [3]. There is convincing evidence that youth centres are not an effective way to reach adolescents with SRH programmes and services, especially female adolescents [7]. Our study therefore focused on a school-based approach.

In-school SRH interventions can be effective if they meet specified standards of quality [8]. They can also increase access for adolescents who need mental health services [9], although they do not always reach those who need them most [10]. After-school SRH programmes, on the other hand, can complement in-school programmes or could serve as an alternative approach if making them available in schools is not feasible. There is evidence that after-school programmes implemented predominantly in the United States have had positive effects on academic, social and emotional outcomes [11–13], although their impact on SRH outcomes has not been assessed sufficiently.

The extent to which adolescents attend and participate in after-school SRH programmes is an important consideration. There is evidence that the greater the number of HIV prevention sessions that adolescents are exposed to, the greater the positive effects on sexual risk behaviour [14]. The potential effectiveness of after-school programmes may therefore depend on the extent to which adolescents, especially those at highest risk for adverse SRH outcomes, are exposed to the sessions. To our knowledge there has been no previous research anywhere in the world on adolescent attendance at after-school SRH programmes. Taking services to places where adolescents congregate, such as schools, has logical appeal, but there is a paucity of evidence on the effects of school-based SRH service provision on adolescent SRH service uptake [7].

It is clearly important to know the extent to which vulnerable adolescents, such as those having an early sexual debut, who have been exposed to IPV and sexual violence, and who are at risk of HIV and STIs [15–17], would attend after-school SRH programmes. Adolescents with mental health problems are also more vulnerable because they are at increased risk for communicable diseases such as HIV and STIs [18]. Furthermore, mental health problems have been associated with poor uptake of sexual and reproductive health care. For example, among people living with HIV, common mental health problems have been associated with poor linkage to care after testing [19], and poor adherence to antiretroviral therapy [20].

Characteristics of an adolescent’s school environment and their experiences of the social and physical aspects of school life can create vulnerability to poor health outcomes or provide resilient environments. Perceived “school connectedness,” one of the commonly used domains of school climate [21], has been shown to be protective against poor mental health, substance use, school drop-out and sexual risk behavior [22, 23]. School climate attributes might therefore facilitate or impede attendance at after-school programmes.

Ensuring attendance of young people at after-school programmes has been shown to be challenging [13]. Demographic factors such as age, sex, migrant status and “race” have been associated with attendance [24]. Except for after-school sport programmes, females are more likely to attend than males, and attendance has been shown to drop with increasing age (possibly mediated by the congruence between the adolescent’s developmental needs and the programme content) [24]. In the United States, “Black” and “Hispanic” youth are less likely to attend (possibly due to social marginalization or lower academic expectations) than “White” youth [24]. There is conflicting evidence about whether young people who are migrants have higher (possibly due to higher academic expectations and optimism for the future) or lower (possibly due to social marginalization) levels of attendance and engagement [24]. A study of students who dropped out of an after-school programme that focused on preventing delinquency were the subgroup with the greatest need and came from the neighborhoods with higher levels of social disorganization [25]. What little evidence there is, suggests that those who may benefit most from after-school programmes may be those least likely to attend.

The PREPARE intervention incorporated an after-school HIV prevention programme held on school premises that aimed to reduce sexual risk behavior, IPV and improve SRH outcomes among young adolescents. Using a cluster randomised controlled trial design [26] we originally intended to evaluate an *in*-school programme delivered as part of the educational curriculum. Due to changing education policies during 2012/3 in the Western Cape however, the research team was prevented from implementing it during school hours. After-school interventions were consistent with the Provincial Government’s policy of structured sport, recreation, arts and culture activities and the PREPARE intervention therefore was incorporated in this way. Given the existing literature from outside the SRH domain, we were concerned whether we would reach the higher-risk adolescents who had had an early sexual debut or who had already been recurrent victims or perpetrators of IPV.

The objectives of the study were to describe the characteristics and risk behaviours of adolescents in the intervention arm of the trial, and the factors associated with their attendance at the after-school programme. We assessed whether we could recruit and retain adolescents especially vulnerable to adverse SRH outcomes: those who reported at baseline that they had already had sex, had been exposed to recurrent IPV and sexual violence, and those with poorer mental health. We investigated whether participants’ perceptions of school climate (including school safety, school connectedness and school physical environment) were associated with programme attendance. We also explored motivations for and barriers to participants’ attendance.

## Methods

### Ethics approval

The study was approved by the Human Research Ethics Committee, Faculty of Health Sciences, University of Cape Town (REC Ref: 268/2010) and by the Western Cape Education Department and the Western Cape Department of Health.

### After-school intervention

The PREPARE intervention included a 21-session, after-school programme based on the Respect4U programme, [27] which was developed and extensively piloted among Grade 8 students in nine public Western Cape High schools over three years. It included students who spoke all three of the main languages in the Province. The sessions were interactive, activity-based and designed to increase motivation and skills which might help to delay sexual debut and increase condom use, change gender norms and power inequities, and improve communication to prevent the use of violence in relationships (Table 1). Each session built on the previous sessions, but participants did not have prior detailed information about the session content before they attended. Sessions were facilitated by staff employed by the PREPARE project, who had been screened for positive gender norm attitudes and comfort with teaching sexeducation and doing condom demonstrations. They received a two-week training and subsequent weekly supervision and session preparation support. We also collaborated with the Western Cape Department of Health, the City of Cape Town Health Department, and the Desmond Tutu HIV Foundation to implement a school health service (SHS) to increase adolescents’ access to sexual and reproductive health services such as condoms, contraception, STI management and treatment and pregnancy tests. This service was offered at schools at the same time as the education sessions, and was modeled on new South African policy [28]. Each PREPARE participant was offered the opportunity of a comprehensive “general health check” by the nurse, but which also included SRH education, screening and referral for SRH problems, and a follow-up consultation if necessary.

**Table 1:** The PREPARE after-school educational programme: session topics and objectives

Topic (number of sessions)	Objectives
Values clarification (1)	Meet facilitator, learn about programme, identify personal values and aspirations including how they want to treat people and be treated
Assertiveness and communication (2)	Identify four communication styles and their consequences
	Practice assertive communication in the context of sexual decision making
Gender and power (2)	Differentiate the concepts ‘sex’ and ‘gender’
	Critically analyses dominant social ideas about gender roles and gender power
	Explore the kind of man or woman they want to be
Relationships (6)	Identify characteristics of a caring relationship
	Identify the qualities they value in an intimate partner
	Identify and learn to respond to relationship problems
	Develop skills to begin and end relationships respectfully and safely
Sexual decision making (4)	Learn about positive and negative consequences of having sex
	Develop action plans to prevent having sex until they are ready
	Identify behaviours that put them at risk of HIV, STIs and pregnancy
	Critically analyse the risks of multiple partnerships, intergenerational partnerships and transactional sex
	Develop skills to use a condom
Violence (4)	Recognise types of IPV and warning signs
	Understand reasons people use violence
	Reflect on own values in relation to violence
	Understand laws related to IPV and sexual violence and the legal support services
	Demonstrate risk monitoring and safety planning skills
Support (1)	Develop understanding and empathy towards victims of IPV and sexual violence
	Understand the importance of seeking help for IPV and sexual violence
	Identify the ways and places to get help, and how to support friends
Creating lasting change (1)	Consolidate and share what they have gained from the programme
	Reflect on their ability to act as agents of change within their school and community

In South Africa, almost all (98.8 %) children aged 7 to 15 years attend school [29]. The study population comprised Grade 8 adolescents attending public high schools in the Western Cape province. The PREPARE trial sample size, (40 high schools with at least 75 participants in each), was calculated for the primary outcomes [26]. They were sampled using the database of 359 public high schools in the Province excluding those with Grade 12 pass rates below 40 % (1 school) indicating their inability to deliver on their core educational mandate, and above 97 % (33 schools) indicating well-resourced schools already able to offer students the types of interventions proposed by PREPARE. We also excluded schools in two of the 8 districts situated far from Cape Town (67 schools), and schools participating in other HIV prevention trials. The analyses presented use longitudinal data from the Grade 8 participants in those schools, randomly allocated to the intervention arm. A statistician, ‘blind’ to the identity of the schools, randomly allocated the 42 sampled schools to intervention or control arms using methods described elsewhere [26].

We invited 6244 students to participate in the PREPARE trial and 3451 (55.3 %) returned signed parental/care-giver consent forms, gave assent and participated in the baseline survey in February and March 2013. The non-responders included 69 students and 281 parents who declined permission for their child to participate and the remainder were students who did not return signed parental consent forms.

To recruit adolescents into the PREPARE RCT, a drama student gave talks to each sampled Grade 8 class, during school hours. She explained the intervention as follows: “*PREPARE is a project which aims, through fun activities, to help young people like you (and me) develop healthy and happy relationships with each other. That’s the sort of relationships that you will be forming from today for the rest of your life, with friends you’ll meet in your class or in your community, a boy or girl that you secretly like sitting two rows in front of you, also the role you have in your family. What PREPARE focuses on is the prevention of violence in these relationships and the prevention of HIV/AIDS*.”

Eligible students were invited to the after-school programme after they had completed the baseline survey. To encourage attendance, we offered refreshments at each after school session and provided small gifts of stationary at selected sessions. Each participant was also provided with a “loyalty card”, which was stamped on attendance at each session or nurse consultation. We gave a R50.00 (~US$5) supermarket gift voucher and certificate to those who attended at least 15 times.

### Measures

The baseline survey was conducted during school hours and included items on sexual risk behaviour developed and tested in a previous HIV prevention RCT [30]. To assess exposure to intimate partner violence, we adapted measures from the WHO survey [31]. Recurrent IPV victimisation (versus an isolated incident or no exposure) was measured by asking whether the participant had been hit, pushed, kicked, choked or burned or forced into sex or threatened with violence by a girlfriend or boyfriend more than once in the past six months. Recurrent IPV perpetration (versus an isolated incident or no perpetration) was measured by asking whether the participant had perpetrated any of these same actions on a girlfriend or boyfriend more than once in the past six months. Sexual violence victimisation was measured by asking whether the participant had ever been forced to have anal or vaginal sex, or had ever been touched sexually against his/her wishes.

As a measure of mental health problems, we included the self-report version of the Strengths and Difficulties Questionnaire (SDQ) [32], which has been designed for 11–17 year olds. It is a behavioural screening questionnaire assessing 25 psychological attributes, both positive and negative. The items comprise 5 sub-scales: emotional symptoms, conduct problems, hyperactivity/inattention, peer relationship problems, and prosocial behaviour [33]. We used the English version and undertook standard procedures for translation and back translation as required by the authors and developers of the SDQ to develop Afrikaans and isiXhosa versions. The translation phase also included a ‘cognitive review’ of the Afrikaans and isiXhosa versions for cultural and pragmatic appropriateness, and to assess whether the translated words and ideas accurately reflected the original version. When there were uncertainties, we contacted the original developer of the scale for clarification. Internal consistency was adequate for the prosocial (Cronbach’s α = 0.69) and emotional symptoms (α = 0.60) sub-scales, but inadequate for the hyperactivity (α = 0.51), conduct problems (α = 0.37), and peer problems (α =0.30) sub-scales. The SDQ was developed to be a multi-dimensional diagnostic tool rather than a scale-based on a reflective measurement model. Using Cronbach’s alpha on the SDQ sub-scales may therefore represent an underestimation of their reliability. For example several other studies have found low estimates of reliability using Cronbach’s alpha, especially for the conduct and peer problem subscales[34–36].) The SDQ has been used previously in South Africa [37, 38] but has not yet received psychometric validation in a South African context. We also included a question about attempted suicide by asking whether, in the past six months students had tried to harm themselves in a way that could have resulted in their death (self-harm).

School climate was assessed by adapting a survey developed by The Safe Communities Safe Schools Program (SCSS), (Center for the Study and Prevention of Violence; http://www.colorado.edu/cspv/; accessed May 2014). The «school safety» subscale comprised 3 items («Some students at my school say they will hit or beat others»; «At my school it is easy for criminals to come into the school ground» and «Students often get hurt at my school»; Cronbach’s Alpha 0.70). The «school connectedness» subscale comprised 4 items («I like school»; «I look forward to going to school»; «I try hard at school»; and «Finishing high school is important to me»; Cronbach’s Alpha: 0.81). The school physical environment sub-scale included 3 items («My school building is clean»; «I like the way my school looks» and «My school is well-maintained»; Cronbach’s Alpha: 0.79).

Subsequent to the baseline survey, we measured participants’ attendance at each of the 21 weekly after-school SRH education sessions over 6 months between February and September 2013, using a register of participants, and roll-calls at each session. School nurses kept registers of the participants who attended the health service provided. Each participant’s attendance record was linked with the data from his or her baseline survey.

A follow-up survey was conducted 6 months after baseline, immediately post-intervention and included two open-ended questions to assess motivators and barriers to attendance. We asked: “If you attended PREPARE sessions after school, please tell us why you decided to come” and “If you did not attend any of the PREPARE sessions after school, please tell us why not”. The responses were coded into themes and analysed quantitatively. Participants could report more than one reason. Of students participating at baseline, 96 % participated in the 6 month follow-up survey.

### Analysis

Means and standard deviations were calculated for continuous and interval data and proportions for categorical data. Chi-square tests and analyses of variance were used to describe the sample. Bivariate and multiple Poisson regression analyses were used to assess which factors were associated with attendance at the 21 after-school sessions. Bivariate and multiple logistic regression analyses were used to assess which factors were associated with visiting the nurse. For the multiple regression models, cases with missing data on variables of interest were excluded (listwise deletion). Participants were clustered within schools, and complex model and mixed model procedures were used to adjust all standard errors for the effect of clustering.

In 18 of the 20 schools, we implemented all 21 educational sessions. In two schools we were not able to implement all sessions. One of these schools was affected by a religious festival which occurred during the implementation period. In the other, after the first few sessions, it became impossible for the school to allocate an afternoon to implement the programme due to other after-school programmes. The 154 participants from these two schools were excluded from analysis. 3451 Grade 8 students participated in the PREPARE trial by completing the baseline assessment and 1576 of them were in the 18 intervention schools and were included in the analyses.

## Results

The students’ average age at baseline was 13.8 years. The majority (94.0 %) were under 16 years of age and 42 % were male. At least one in five (22.7 %) reported that they had ever had vaginal, anal or oral sex (Table 2). Among those participants who reported at baseline that they had ever had sex (*N* = 358), 203 (56.7 %) were younger than 15 years old, and 295 (82.4 %) were younger than 16 years old. Many of the participants (31.7 %) reported that they had been a victim of sexual violence; 16.1 % had been a victim of physical, emotional or sexual IPV and 10.3 % had been a perpetrator of IPV. One in eight (15.7 %) reported having attempted suicide during the six months prior to the baseline survey.

**Table 2:** Characteristics of 1576 adolescents and their attendance at the after-school sexual and reproductive health programme

	N	Total sample	A: Non-attenders (0 % of education sessions) (*N* = 336)	B: Low attenders (less than 50 % of education sessions) (*N* = 653)	C: High attenders (50 % or more of education sessions) (*N* = 587)	Significant differences
Gender (Male %)	1534	42.0 % (644)	53.7 % (176)	42.1 % (268)	35.1 % (200)	A > B > C
Age (Mean; SD)	1539	13.77 (1.01)	13.94 (1.08)	13.83 (1.01)	13.60 (0.94)	A, B > C
SES^a^ (Mean; SD)	1576	5.87 (1.67)	6.06 (1.51)	5.76 (1.78)	5.87 (1.62)	ns
How well did you do in school?^b^	1532	3.95 (0.92)	3.87 (0.93)	3.96 (0.92)	3.97 (0.91)	ns
Have you ever repeated a school year? (% yes)	1486	23.6 % (351)	33.4 % (105)	24.8 % (150)	17.0 % (96)	A > B > C
Delinquency^c^	1536	9.2 % (142)	12.8 % (42)	9.8 % (62)	6.6 % (38)	A, B > C
Drug use^d^	1576	3.3 % (52)	5.4 % (18)	3.5 % (23)	1.9 % (11)	A > C
Did you bully someone at school in the past 6 months? (% yes)	1451	13.4 % (195)	19.7 % (62)	13.5 % (80)	9.7 % (53)	A > B > C
Were you bullied by someone at school in the past 6 months? (% yes)	1390	19.7 % (275)	18.4 % (56)	20.6 % (116)	19.7 % (103)	ns
Self-harm (% yes)	1302	15.7 % (204)	20.7 % (56)	16.6 % (86)	12.1 % (62)	A, B > C
Knowledge of HIV/condoms (mean; SD)^e^	1553	0.42 (0.20)	0.41 (0.19)	0.43 (0.21)	0.42 (0.20)	ns
Ever had oral, anal or vaginal sex	1576	22.7 % (358)	27.4 % (92)	26.5 % (173)	15.8 % (93)	A, B > C
Age of sexual debut (mean years)^i^	198	13.93 (1.83)	14.14 (1.63)	13.90 (1.78)	13.84 (2.26)	ns
Number of sexual partners (mean)^i^	174	3.37 (2.78)	3.29 (2.71)	3.63 (2.84)	2.83 (2.72)	ns
Condom use consistency (% always)^i^	269	19.7 % (55)	17.6 % (12)	20.7 % (28)	19.7 % (13)	ns
Victim of sexual violence (% yes)	1442	31.7 % (503)	34.2 % (107)	36.0 % (210)^A^	27.7 % (151)	A, B > C
Victim of IPV (% yes)	1452	16.1 % (259)	16.8 % (53)	13.8 % (82)	10.2 % (55)	A > C
Perpetrator of IPV (% yes)	1427	10.3 % (163)	12.3 % (38)	11.1 % (65)	5.3 % (28)	A, B > C
School safety^f^	1459	2.90 (1.06)	2.77 (1.06)	2.89 (1.07)	2.98 (1.05)	A < C
School connectedness^f^	1467	4.56 (0.67)	4.47 (0.73)	4.53 (0.69)	4.64 (0.60)	A, B < C
School appearance^f^	1475	3.93 (1.02)	3.88 (1.06)	3.93 (1.02)	3.94 (1.00)	ns
Emotional symptoms^g^	1549	3.89 (2.39)	3.67 (2.21)	4.02 (2.47)	3.88 (2.40)	A < B
Conduct problems^g^	1546	2.39 (1.75)	2.47 (1.78)	2.51 (1.85)	2.19 (1.61)	A, B > C
Hyperactivity scale^g^	1547	2.70 (2.01)	2.95 (2.04)	2.78 (2.01)	2.47 (1.97)	A, B > C
Peer problems scale^g^	1545	2.77 (1.93)	2.90 (1.86)	2.79 (1.94)	2.67 (1.96)	ns
Prosocial scale^g^	1556	7.82 (2.16)	7.26 (2.32)	7.77 (2.19)	8.21 (1.94)	C > B > A
Number of sessions attended (mean; SD)	1576	8.02 (7.44)	0 (0)	4.26 (2.87)	16.80 (3.12)	C > B > A
Exposure^h^	1576	0.38 (0.35)	0 (0)	0.20 (0.14)	0.80 (0.15)	C > B > A
Visited a nurse (% yes)	1455	17.3 % (252)	0 % (0)	11.6 % (72)	35.6 % (180)	C > B > A

*IPV* intimate partner violence (physical, emotional and/or sexual)

^a^Scale from ‘no household items’ (0) to ‘8 household items’

^b^Scale from ‘Worst of class’ (1) to ‘Best of class (5)

^c^Monthly or more frequent involvement in fighting, stealing or vandalism during the previous 3 months

^d^Monthly or more frequent user of dagga, tic or glue during previous 3 months

^e^Scale from ‘no questions correct’ (0) to ‘all questions correct’ (1)

^f^Scale from ‘Totally disagree’ (1) to ‘Totally agree’ (5) with a higher score indicating more connectedness, perceptions of safety, a better school appearance

^g^Scale from ‘Low’ (0) to ‘high’ (10)

^h^Scale from ‘no attendance’ (0) to ‘full attendance’ (1)

^i^Denominator is those students who reported to ever had had vaginal, anal or oral sex

The mean number of education sessions attended was 8.02 (SD: 7.44; range 0 to 21). Among girls it was 8.8 sessions (SD 7.5) and among boys 6.9 (SD 7.2). Our records showed that 272 females (30.6 %) and 141 males (21.9 %) had attended more than 15 sessions. Three hundred and sixty-three (40.8 %) females and 330 (51.2 %) males attended less than 5 sessions. Participants who reported in the baseline survey that they had ever had sex had a lower rate of attendance than those who did not report having had sex (6.3 vs 8.5, *t*(1574) = 5.17, *p* < .001). Those who reported having been a victim of sexual violence and IPV had lower rates of attendance than those not reporting these experiences (sexual violence: 7.0 vs 8.6, *t*(1440) = 4.08, *p* < .001; IPV: 6.5 vs 8.2, *t*(1450) = 3.22, *p* = .001). Those reporting having perpetrated IPV had a lower attendance rate than those who had not (5.2 vs 8.3, *t*(1425) = 5.44, *p* < .001).

Where participants had attended 50 % or more of the sessions, we assumed they would have attended the core sessions dealing with sexual debut, condoms, and STI and pregnancy prevention. The bivariate analyses (Table 2) revealed that the factors associated with lower attendance (either having attended none or less than 50 % of sessions versus having attended 50 % or more) were being male, older, having repeated a year of school, and reporting delinquent behaviours, drug use, bullying others, having ever had sex, being a victim of sexual violence, being a victim of IPV, and being a perpetrator of IPV. The school climate factors inversely associated with lower attendance were perceptions of school safety and feelings of connectedness to school. The mental health factors associated with lower attendance were higher scores on the conduct problems and hyperactivity SDQ sub-scales, lower scores on the prosocial SDQ sub-scale, and having tried to harm themselves in a way that could have resulted in their death (self-harm).

In multiple Poisson regression where the dependent variable was the number of after-school sessions attended (rate of attendance), adjusting for all other variables, the factors associated with a lower rate of attendance were being male, older, having repeated a school year, reporting delinquent behaviours, having attempted self-harm, ever having had sex, and being a perpetrator of IPV (Table 3). The factors associated with higher attendance were having been bullied at school, being a victim of IPV, reporting a greater feeling of school connectedness, and scoring higher on the SDQ prosocial sub-scale (Table 3). The association between being a victim of IPV and rate of attendance was negative in bivariate analyses, but positive in the multivariate analyses, suggesting a suppression effect. Subsequent in-depth analyses showed this was caused by multiple variables related to sex or violence (i.e. ever having had sex, gender, IPV perpetration and reporting having been a victim of sexual violence).

**Table 3:** Factors associated with the rate of attendance at 21 PREPARE after-school education sessions

	Bivariate regression		Multiple regression^h^(*N* = 986)	
	B	95 % CI	B	95 % CI
Gender (0 = male; 1 = female)	0.25*	0.22 – 0.29	0.16*	0.11 – 0.21
Age	−0.16*	−0.18 – -0.13	−0.08*	−0.11 – -0.05
SES^a^	0.01	−0.01 – 0.02	0.01	−0.01 – 0.02
How well did you do in school?^b^	0.02	−0.01 – 0.04	−0.02	−0.04 – 0.01
Have you ever repeated a school year? (No = 0; Yes = 1)	−0.27*	−0.32 – -0.22	−0.08*	−0.15 – -0.02
Delinquency (No = 0; Yes = 1)^c^	−0.20*	−0.27 – -0.13	−0.11*	−0.21 – -0.01
Drug use(No = 0; Yes = 1)^d^	−0.31*	−0.43 – -0.19	0.10	−0.07 – 0.28
Bullied someone at school in the past 6 months	−0.13*	−0.18 – -0.09	−0.02	−0.08 – 0.04
Was bullied by someone at school in the past 6 months	0.01	−0.03 – 0.05	0.12*	0.07 – 0.16
Self-harm (No = 0; Yes = 1)	−0.24*	−0.30 – -0.18	−0.20*	−0.26 – -0.13
Knowledge of HIV/condoms^e^	−0.08	−0.17 – 0.01	0.10	−0.02 – 0.21
Had ever had vaginal, anal or oral sex (No = 0; Yes = 1)	−0.30*	−0.34 – -0.25	−0.17*	−0.23 – -0.11
Victim of sexual violence (No = 0; Yes = 1)	−0.16*	−0.20 – -0.12	0.01	−0.05 – 0.06
Victim of IPV (No = 0; Yes = 1)	−0.19*	−0.25 – -0.13	0.14*	0.06 – 0.23
Perpetrator of IPV (No = 0; Yes = 1)	−0.45*	−0.53 – -0.37	−0.26*	−0.38 – -0.14
School safety^f^	0.02	−0.01 – 0.04	−0.01	−0.03 – 0.02
School connectedness^f^	0.16*	0.13 – 0.19	0.11*	0.07 – 0.15
School appearance^f^	0.02*	0.01 – 0.04	−0.01	−0.03 – 0.01
Emotional symptoms^g^	0.01	−0.01 – 0.01	−0.01	−0.02 – 0.01
Conduct problems^g^	−0.05*	−0.06 – -0.04	−0.01	−0.02 – 0.01
Hyperactivity scale^g^	−0.03*	−0.04 – -0.02	−0.01	−0.02 – 0.01
Peer problems scale^g^	−0.01	−0.02 – 0.01	0.01	−0.01 – 0.02
Prosocial scale^g^	0.05*	0.04 – 0.06	0.02*	0.01 – 0.03

*IPV* intimate partner violence (physical, emotional and/or sexual)

^a^Scale from ‘no household items’ (0) to ‘8 household items (8)

^b^Scale from ‘Worst of class’ (1) to ‘Best of class (5)

^c^Monthly or more frequent involvement in fighting, stealing or vandalism during the previous 3 months

^d^Monthly or more frequent user of dagga, tic or glue during previous 3 months

^e^Scale from ‘no questions correct’ (0) to ‘all questions correct’ (1)

^f^Scale from ‘Totally disagree’ (1) to ‘Totally agree’ (5) with a higher score indicating more connectedness, perceptions of safety, a better school appearance

^g^Scale from ‘Low’ (0) to ‘high’ (10)

^h^Adjusted for all covariates shown

*p < 0.05

The PREPARE school nurse was visited by 17.3 % of the trial participants in intervention schools, (14.9 % of boys and 18.7 % of girls). Those who attended a greater number of after-school SRH educational sessions were more likely to visit the nurse (Table 2);visiting the nurse was conditional on attendance of at least one education session. In bivariate analyses, being younger, reporting having been bullied, and scoring higher on the prosocial SDQ sub-scale were associated with a significantly greater odds of visiting the nurse (Table 4). In a multiple logistic regression being female, having been bullied in the past 6 months, and having better knowledge about HIV and condoms were associated with a greater odds of visiting the nurse, when adjusting for all other factors (Table 4). The results were similar when students who did not attend any sessions were excluded from the analyses (results not shown). In addition, we explored whether these results would differ for boys and girls. Examination of the stratified results for attendance at education sessions showed subtle differences. Among girls, ever having had sex was not significantly associated with attendance, but a more negative school appearance was (B = −0.04; p < 0.01). Among boys, school performance and reported self-harm was not significantly associated with attendance. Reporting more peer problems, however, was significantly associated with attendance among boys (B = 0.04; *p* < 0.01). Similarly, for visits to the nurse, stratified results showed that, among girls, knowledge about HIV/AIDS and condoms was the only predictor of having visited a nurse (OR = 6.04; *p* < 0.01). Among boys, being a victim of bullying was the only significant predictor of having visited a nurse (OR = 2.98; *p* < 0.001). To confirm whether these observed differences between boys and girls were statistically significant, moderation analyses were needed. Yet, power analysis indicated insufficient power to detect moderation effects (i.e. sample size needed at an expected effect size of f^2^ = 0.02 is approximately 2000) [39].

**Table 4:** Factors associated with attendance at PREPARE school health service

	Bivariate regression		Multiple regression (*N* = 899)^h^	
	OR	95 % CI	OR	95 % CI
Gender (Male = 0; female = 1)	1.35	0.99 – 1.84	1.71*	1.11 – 2.64
Age	0.75*	0.63 – 0.89	0.83	0.64 – 1.06
SES^a^	1.03	0.93 – 1.14	1.01	0.88 – 1.17
How well did you do in school?^b^	1.16	0.98 – 1.37	1.03	0.83 – 1.29
Have you ever repeated a school year? (No = 0; Yes = 1)	0.75	0.51 – 1.09	1.19	0.69 – 2.04
Delinquency (No = 0; Yes = 1)^c^	0.82	0.48 – 1.39	1.02	0.59 – 1.75
Drug use (No = 0; Yes = 1)^d^	1.27	0.58 – 2.82	2.17	0.56 – 8.51
Did you bully someone at school in the past 6 months?	1.00	0.70 – 1.43	0.97	0.58 – 1.62
Were you bullied by someone at school in the past 6 months?	1.53*	1.16 – 2.02	1.88*	1.28 – 2.74
Self-harm (No = 0; Yes = 1)	1.09	0.72 – 1.66	0.90	0.52 – 1.55
Knowledge of HIV/condoms^e^	1.73	0.84 – 3.59	3.23*	1.22 – 8.57
Had ever had vaginal, anal or oral sex (No = 0; Yes = 1)	0.97	0.66 – 1.42	0.93	0.54 – 1.62
Victim of sexual violence (No = 0; Yes = 1)	1.15	0.83 – 1.59	1.01	0.64 – 1.60
Victim of IPV (No = 0; Yes = 1)	1.33	0.85 – 2.06	1.69	0.85 – 3.36
Perpetrator of IPV (No = 0; Yes = 1)	1.07	0.61 – 1.87	1.31	0.54 – 3.18
School safety^f^	0.89	0.77 – 1.04	0.90	0.73 – 1.09
School connectedness^f^	1.24	0.96 – 1.60	1.48	0.99 – 2.18
School appearance^f^	0.95	0.81 – 1.11	0.91	0.75 – 1.12
Emotional symptoms^g^	1.05	0.98 – 1.11	0.98	0.90 – 1.08
Conduct problems^g^	0.93	0.85 – 1.01	0.90	0.79 – 1.04
Hyperactivity scale^g^	1.01	0.94 – 1.08	1.08	0.97 – 1.21
Peer problems scale^g^	1.04	0.96 – 1.12	1.01	0.90 – 1.13
Prosocial scale^g^	1.11*	1.03 – 1.19	1.08	0.97 – 1.21

*IPV* intimate partner violence (physical, emotional and/or sexual)

^a^Scale from ‘no household items’ (0) to ‘8 household items (8)

^b^Scale from ‘Worst of class’ (1) to ‘Best of class (5)

^c^Monthly or more frequent involvement in fighting, stealing or vandalism during the previous 3 months

^d^Monthly or more frequent user of dagga, tic or glue during previous 3 months

^e^Scale from ‘no questions correct’ (0) to ‘all questions correct’ (1)

^f^Scale from ‘Totally disagree’ (1) to ‘Totally agree’ (5) with a higher score indicating more connectedness, perceptions of safety, a better school appearance

^g^Scale from ‘Low’ (0) to ‘high’ (10)

^h^Adjusted for all covariates shown

*p < 0.05

Among participants who attended at least one educational session (*N* = 1240), 570 (45.9 %) responded to the question about motivations to attend. The most common motivators were to gain new knowledge(291; 23.5 %), having a positive attitude towards the intervention (116; 9.4 %), and a wish to receive life coaching (114; 9.2 %). One participant said:

“I needed some information how to handle relationships”.

Another:

“It felt like they understood us as teens”;

“No-one teaches us things in such a fun way”

On asking why s/he attended one participant said:

“To guide me through my life: it helps us be stronger and better people”.

The most common barriers to attendance reported by those who did not attend any sessions (*N* = 336) were structural such as the unavailability of safe transport home (33; 9.8 %). Other barriers included domestic responsibilities such as housework, child care and looking after sick family members (23; 6.8 %), and competing academic and extra-curricular demands such as school work and sports (13; 3.9 %). Only two participants cited negative attitudes about the intervention as the reason they did not attend (2; 0.6 %). These barriers were also the most commonly reported reasons for non-attendance of sessions among those who attended at least one session and not all sessions.

## Discussion

It is critical to provide young adolescents in sub-Saharan Africa with access to proven-effective HIV prevention interventions such as comprehensive sex education and SRH services including access to condoms, contraception and HIV testing [40]. However access to such SRH education and services is often reported to be inadequate [40]. In-school, teacher-implemented, curriculum-based HIV prevention programmes are important because they have the potential to have a broad reach, but poor fidelity has been reported especially related to important topics such as condoms [41, 42]. After-school SRH programmes like PREPARE have the potential to reach and assist adolescents to make informed choices about sexual activity and to choose more gender-equitable intimate relationships [27], to access condoms, contraception, HIV testing and other health resources.

We investigated the HIV-risk profile of non-attenders, attenders and high attenders and found that considering important hypothesized risk markers, higher risk profiles were associated with lower rates of attendance of SRH education sessions. Fewer sessions were attended by adolescents who had already had their sexual debut, compared with those who had not, and by perpetrators of IPV compared to non-perpetrators Fewer sessions were accessed by participants with poorer scores on the mental health assessment and who had attempted self-harm. Fewer sessions were also attended by adolescents who felt less connected to school. Two of our findings contradicted this pattern. Boys who experienced more peer problems had higher attendance rates, possibly because the sessions were perceived as helpful in this domain. Adolescent females, who in our setting are at higher risk of HIV, IPV and sexual violence than their male contemporaries [2, 43, 44], were more likely than males to attend the education sessions. Among girls in our sex-stratified analyses there was no difference in attendance rates between those who had and those who had not ever had sex, indicating that for this variable, after-school SRH programmes are equally accessible to girls with a higher risk profile. This is an important finding given that young women have been found to be less likely to access other out-of-health facility SRH services such as youth clubs [7]. After-school SRH programmes therefore have the potential to improve adolescent girls’access to SRH services.

It is important to note that we observed relatively small differences in attendance rates: vulnerable adolescents attended only approximately 2 fewer sessions than those not defined as vulnerable. However, we found a relatively low overall rate of attendance (an average attendance of fewer than half the sessions). Negative evaluations of the intervention were rarely expressed as reasons for non-attendance. Instead structural barriers related to transport and competing responsibilities were key. A common reason for drop-out from after-school social and educational programmes is that the young people have found them boring [24], but this does not appear to apply in our study. Our findings suggest that the implementation of arrangements to facilitate safe transport home would have overcome many of the barriers to attendance. More extensive communication with families about the programme might lead to adolescents being released from domestic responsibilities. This highlights the importance of focusing on community acceptance of the intervention. Community-based information, education and communication has been shown to increase SRH service use among adolescents [7], and is likely to be an appropriate way to increase adolescent uptake of after-school programmes.

Interestingly the PREPARE after-school health service was attended equally by adolescents across risk profiles, albeit at relatively low rates. This suggests there may be a key role for school nurses as providers of SRH and mental health education, screening and referral for care. The school health service was accessed more by those who had been bullied in the past 6 months compared with those who had not been bullied, and by those with better knowledge about HIV and condoms, possibly indicating that they sought it out for preventive and support services. In our trial, access to the health service was conditional on attending after-school educational sessions. In other settings, alternative systems for offering adolescents appointments for health care services might increase access, but there is little evidence to guide the design of such arrangements [10].

Apart from removing the structural barriers to attendance it has been hypothesized that engagement in after-school programmes is facilitated by an atmosphere that is caring and supportive, rather than cold and critical [24] and our finding regarding the association between school-connectedness and attendance bears this out. Thus interventions to improve the school climate and foster school-connectedness are likely to be important in settings (such as South Africa) where schools can be unsafe spaces for adolescents. Strategies such as adolescent participation in programme design might be particularly important to generate demand among adolescents and might be an effective way to increase attendance and to ensure that the content is valued.

There is a dearth of evidence about improving vulnerable adolescent populations’ access to SRH services and programmes, whether provided in schools or elsewhere [7]. Vulnerable adolescents may need to be reached through a combination of in-school programmes, programmes at an early age, or through alternative service delivery modalities such as single-session, one-on-one interventions [45]. In addition, we need to consider opportunities to provide care and support for adolescents with mental health problems and neurodevelopmental academic problems.

Our study had several limitations. Our findings do not represent the whole of the Western Cape Province because of the exclusion of two educational districts. Further only 55.3 % of sampled adolescents had signed parental consent forms and assented to take part in the study. Adolescents without signed parental consent might have been at higher risk than those with parental consent. We did not measure adolescents’ engagement with and participation in the session activities directly, which have been proposed as predictors of beneficial outcomes [24, 46]. Not all adolescents in the sample had the opportunity to visit the school nurse. In some schools the demand for her services could not be met. We did not collect data to determine whether the way the nurse provided the service might have contributed to the low uptake of the service. This needs to be investigated in future studies. More than half of the participants did not answer the open-ended questions about their motivations to attend or reasons they did not attend. Measurement error in three of the SDQ sub-scales might have attenuated associations between these aspects of mental health and attendance. Our intervention was piloted as an in-school programme [27]. Upon redesigning it for after-school, we implemented a set of measures to overcome barriers to attendance including the provision of food and incentives. We had hoped that participation incentives would have a differential effect, motivating the most vulnerable adolescents to attend the after-school program. Cash and other incentives are sometimes used to promote adolescent uptake of preventive health services [47] and there is some evidence that they have sustained adolescents’ attendance of after-school programmes focusing on outcomes other than health, but the effects have been inconsistent across studies and none of the evidence is related to after-school SRH programmes [24]. All adolescents in the intervention arm were offered incentives so we were not able to assess the extent to which the incentives increased attendance, and whether they were more effective for more vulnerable adolescents. However access was equitable across levels of socio-economic status.

## Conclusions

We still need to find ways to improve adolescent access to and attendance at comprehensive SRH education and effective SRH services. Services in health facilities need to be complemented by out-of-facility interventions. With efforts to reduce the structural barriers to attendance and increase community support of such programmes, after-school SRH interventions could be one of the means to reach adolescents and in particular to reach adolescent girls. Our study has shown that adolescents were very positively disposed to such programmes, and valued the education, guidance and support they provided. However, it will be important for future studies to address the specific barriers to attendance experienced by more vulnerable adolescents, so they can be attracted and retained in after-school SRH programmes.

## Abbreviations

SRH: Sexual and reproductive health
IPV: Intimate partner violence
RCT: Randomised controlled trial
